# Efficacy and safety of Chinese herbal acupoint application for post-stroke sleep disorders: a systematic review and meta-analysis

**DOI:** 10.3389/fneur.2026.1881026

**Published:** 2026-09-10

**Authors:** Yujing Xu, Yuanjing Xia, Xiaoyu Wang, Xianxian Qian, Yue Xiang, Tanfang Wei, Zhongxia Fu

**Affiliations:** 1 School of Nursing, Gansu University of Chinese Medicine, Lanzhou, China; 2 School of Nursing, Northwest Minzu University, Lanzhou, China; 3 Department of Neurology, Gansu Provincial Maternity and Child Health Care Hospital (Gansu Provincial Central Hospital), Lanzhou, China

**Keywords:** acupoint application, meta-analysis, post-stroke insomnia, sleep disorders, stroke, systematic review, traditional Chinese medicine

## Abstract

**Objective:**

To evaluate the efficacy and safety of Chinese herbal acupoint application for post-stroke sleep disorders, particularly insomnia and poor sleep quality, and to provide evidence for sleep management during post-stroke rehabilitation.

**Methods:**

This systematic review and meta-analysis followed the PRISMA 2020 reporting guideline and was registered in PROSPERO (CRD420261358357). China National Knowledge Infrastructure, Wanfang Data, VIP Database, Chinese Biomedical Literature Database (SinoMed), PubMed, the Cochrane Library, Embase, and Web of Science were searched from inception to April 1, 2026. Randomized controlled trials comparing acupoint application plus a comparator intervention with the same comparator intervention alone were included. Two reviewers independently screened studies and extracted data. Risk of bias was assessed using the original Cochrane Risk of Bias tool (RoB 1), certainty of evidence was evaluated using GRADE, and meta-analyses were performed in RevMan 5.4.1.

**Results:**

Seven randomized controlled trials involving 608 participants were included. Compared with the matched comparator intervention alone, adjunctive acupoint application was associated with a higher trial-defined clinical response rate [odds ratio (OR) = 3.50, 95% confidence interval (CI) 2.05–5.97; *p* < 0.00001] and lower Pittsburgh Sleep Quality Index scores [mean difference (MD) = −2.69, 95% CI − 3.84 to −1.55; *p* < 0.00001], Hamilton Anxiety Rating Scale scores (MD = −9.38, 95% CI − 10.09 to −8.68; *p* < 0.00001), and Hamilton Depression Rating Scale scores (MD = −7.58, 95% CI − 9.08 to −6.08; *p* < 0.00001). Only two studies reported adverse events, and no study reported post-treatment follow-up. The certainty of evidence was low for trial-defined clinical response and very low for the other outcomes.

**Conclusion:**

Low- to very-low-certainty evidence suggests that adjunctive Chinese herbal acupoint application may improve subjective sleep quality and emotional symptoms after stroke. However, the true effects may differ substantially from the pooled estimates. Current evidence is insufficient to establish safety or sustained benefit; larger, multicenter, rigorously designed trials are required.

**Systematic review registration:**

https://www.crd.york.ac.uk/prospero/, CRD420261358357

## 1 Introduction

Stroke is a leading cause of death, disability, and long-term care needs worldwide. As acute treatment and secondary prevention have improved, an increasing number of stroke survivors enter rehabilitation and chronic recovery, during which sleep disorders are common and clinically important non-motor complications. Post-stroke sleep disorders include insomnia, sleep-disordered breathing, excessive daytime sleepiness, circadian rhythm disorders, restless legs syndrome, and rapid eye movement sleep behavior disorder. Insomnia and poor sleep quality are particularly common during rehabilitation and may impair daytime functioning, participation in rehabilitation, emotional stability, cognitive recovery, and quality of life (1–7).

Current management strategies include sleep-hygiene education, environmental modification, psychological support, pharmacotherapy, rehabilitation, and complementary interventions such as acupuncture, auricular acupressure, and acupoint application (8–10). Chinese herbal acupoint application is an external traditional Chinese medicine treatment in which herbal preparations are applied to specific acupoints to provide local stimulation and transdermal delivery of medicinal constituents. The intervention is noninvasive, relatively simple to administer, and generally acceptable to patients, and may therefore be suitable as an adjunct to nursing and rehabilitation care. However, available trials vary substantially in design, treatment intensity, herbal composition, acupoint selection, co-interventions, and outcome reporting, leaving its clinical value for post-stroke sleep disorders uncertain. We therefore conducted a systematic review and meta-analysis to evaluate the efficacy and safety of acupoint application for post-stroke sleep disorders.

## 2 Materials and methods

### 2.1 Registration and reporting

This systematic review was registered in PROSPERO (CRD420261358357) and is reported in accordance with the PRISMA 2020 statement (11).

### 2.2 Eligibility criteria

Eligibility criteria were developed using the Population, Intervention, Comparator, Outcomes, and Study design (PICOS) framework (Table 1). Eligible participants had a confirmed diagnosis of stroke and a concurrent sleep disorder. Stroke was diagnosed according to recognized cerebrovascular disease guidelines (12), and sleep disorders were confirmed by clinical assessment or a validated sleep-related instrument. Clinical responses reported by the original trials were accepted according to each trial’s own definition and were uniformly described as “trial-defined clinical response.” The exact response criteria and numbers of events in each group were also extracted to enable transparent comparison across studies.

**Table 1:** PICOS eligibility framework.

PICOS element	Eligibility criterion
Population	Patients with a confirmed diagnosis of stroke and a concurrent sleep disorder. Stroke was diagnosed according to recognized cerebrovascular disease guidelines, and the sleep disorder was confirmed by clinical assessment or a validated sleep-related instrument.
Intervention	Chinese herbal acupoint application added to the comparator intervention.
Comparator	Conventional medical treatment, rehabilitation training, acupuncture, psychological nursing, or Chinese herbal medicine.
Outcomes	Trial-defined clinical response, PSQI, HAMA, HAMD, and adverse events.
Study design	Randomized controlled trial.
Other criteria	Provided baseline characteristics were comparable, no restriction was imposed on sex, age, stroke type, or traditional Chinese medicine syndrome pattern.

### 2.3 Exclusion criteria

Reports were excluded if participants had major coexisting diseases, the publication duplicated another report, sufficient data could not be extracted, or the report was a conference abstract or conference paper. The review protocol prespecified inclusion only of randomized controlled trials published as complete, peer-reviewed journal articles. Master’s and doctoral theses were therefore excluded to adhere to the prespecified criteria and to reduce the risk of duplicate counting if a thesis was subsequently published as a journal article. We acknowledge that this decision may have omitted unpublished or negative findings and may have increased the risk of publication bias.

### 2.4 Information sources and search strategy

China National Knowledge Infrastructure (CNKI), Wanfang Data, VIP Database, the Chinese Biomedical Literature Database (SinoMed), PubMed, the Cochrane Library, Embase, and Web of Science were searched from inception to April 1, 2026. Search concepts covered stroke, cerebral infarction, cerebral hemorrhage, cerebrovascular accident, sleep disorder, insomnia, hypersomnia, and acupoint application. Intervention-related terms included “acupoint application,” “acupoint sticking,” “acupoint plaster,” and “herbal plaster.” Formal citation tracking was not performed, trial registries were not searched, and gray literature was not systematically searched. These limitations, together with the exclusion of theses, may have reduced search completeness and increased the risk of publication bias. The PubMed search strategy is shown in Table 2. Complete reproducible search strategies for all databases are provided in Supplementary file 1, including the platform, fields, full syntax, limits, and final search date for each database.

**Table 2:** PubMed search strategy.

Search set	Search string
#1	“Stroke”[MeSH Terms] OR stroke[Title/Abstract] OR apoplexy[Title/Abstract] OR “brain infarction”[Title/Abstract] OR “cerebral infarction”[Title/Abstract] OR “cerebral ischemia”[Title/Abstract] OR “cerebral embolism”[Title/Abstract] OR “cerebral hemorrhage”[Title/Abstract] OR “cerebrovascular accident*”[Title/Abstract] OR “cerebrovascular disorder*”[Title/Abstract]
#2	“Sleep Wake Disorders”[MeSH Terms] OR “sleep disorder*”[Title/Abstract] OR insomnia[Title/Abstract] OR “difficulty sleeping”[Title/Abstract] OR hypersomnia[Title/Abstract] OR “sleep disturbance*”[Title/Abstract]
#3	“acupoint application”[Title/Abstract] OR “acupoint sticking”[Title/Abstract] OR “acupoint plaster”[Title/Abstract] OR “herbal plaster”[Title/Abstract]
#4	#1 AND #2 AND #3

### 2.5 Study selection and data extraction

Duplicates were removed using EndNote 20. No machine-learning or other automated screening tool was used beyond EndNote 20 for reference management and deduplication. Two reviewers independently screened titles, abstracts, and full texts against the prespecified eligibility criteria and extracted the first author, sample size, age, disease duration, intervention and comparator, treatment frequency and duration, outcomes, and adverse events. The revised extraction framework additionally captured stroke stage, diagnostic criteria for the sleep disorder, baseline sleep severity, selected acupoints, herbal composition, rationale for treatment selection, follow-up duration, adverse-event monitoring procedures, and the exact criteria and event counts for trial-defined clinical response. For the Hamilton Anxiety Rating Scale (HAMA) outcome, the reviewers separately verified group assignment, endpoint means, standard deviations, sample sizes, number and specific version of scale items, item-level scoring range, scoring direction, assessment time point, rater training, and inter-rater reliability. Both reviewers cross-checked extracted data against the text and tables of the original reports. Original study authors were not contacted for incomplete information; such items were recorded as not reported (NR) without imputation. Disagreements were resolved through discussion and, when necessary, consultation with a third reviewer.

### 2.6 Risk-of-bias assessment

Two reviewers independently assessed methodological quality using the original domain-based Cochrane Risk of Bias tool (RoB 1). Domains included random-sequence generation, allocation concealment, blinding of participants and personnel, blinding of outcome assessment, incomplete outcome data, selective reporting, and other potential sources of bias. Each domain was rated as low, high, or unclear risk.

### 2.7 Certainty-of-evidence assessment

Two reviewers independently used the Grading of Recommendations Assessment, Development and Evaluation (GRADE) approach to assess the certainty of evidence for each major outcome. Evidence from randomized controlled trials initially started at high certainty and could be downgraded for risk of bias, inconsistency, indirectness, imprecision, or suspected publication bias. Indirectness was assessed by considering the correspondence between the review question and study populations, intervention and co-interventions, comparators, outcome definitions, and assessment time points. Imprecision was judged primarily according to whether the 95% CI crossed the null or a clinically meaningful effect range, rather than solely according to the number of studies. Because fewer than 10 studies contributed to every outcome, publication bias was not formally assessed using funnel plots or regression tests. Instead, it was judged qualitatively from the size of the evidence base, geographic setting and center type, the concentration of small studies and positive results, and whether trial registries and gray literature had been searched. Outcome-specific reasons for downgrading are provided in Table 3 and its footnotes. Disagreements were resolved by consensus. Certainty was categorized as high, moderate, low, or very low (13).

**Table 3:** GRADE summary of the certainty of evidence.

Outcome	Studies/participants	Effect estimate	Certainty of evidence	Main reasons for downgrading
Trial-defined clinical response	5/466	OR 3.50 (95% CI 2.05–5.97)	Low	Serious risk of bias; suspected publication bias [a]
PSQI	6/536	MD − 2.69 (95% CI − 3.84 to −1.55)	Very low	Serious risk of bias; serious inconsistency (I^2^ = 95%); suspected publication bias [b]
HAMA	2/182	MD − 9.38 (95% CI − 10.09 to −8.68)	Very low	Serious risk of bias; serious indirectness; suspected publication bias [c]
HAMD	2/182	MD − 7.58 (95% CI − 9.08 to −6.08)	Very low	Serious risk of bias; serious inconsistency (I^2^ = 81%); suspected publication bias [d]
Adverse events	2 studies	Narrative synthesis only	Very low	Serious risk of bias; very serious imprecision; incomplete monitoring and reporting [e]

GRADE definitions: High certainty, the true effect is very likely to be close to the effect estimate; moderate certainty, the true effect is likely to be close to the estimate but may be substantially different; low certainty, confidence in the effect estimate is limited; very low certainty, there is very little confidence in the effect estimate.

[a] Trial-defined clinical response was downgraded one level for serious risk of bias because allocation concealment and blinding were absent or inadequately reported. Although background treatments, co-interventions, and response definitions differed, each trial used a matched background treatment within the study to estimate the added effect of acupoint application; the outcome was therefore not separately downgraded for indirectness. The evidence base was small, all studies were published in Chinese, most were small single-center studies with positive results, and neither trial registries nor gray literature was searched; the outcome was downgraded one level for suspected publication bias. [b] PSQI was downgraded one level each for serious risk of bias due to absent or inadequately reported allocation concealment and blinding, and for serious inconsistency (I^2^ = 95%). The evidence comprised few, mainly small single-center studies with generally positive findings, and trial registries and gray literature were not searched; the outcome was downgraded one level for suspected publication bias. [c] Both studies used the 14-item HAMA with the same scoring direction, but the specific Chinese translation or revision, rater training, and inter-rater reliability were not reported; endpoint assessments occurred at 2 and 4 weeks and co-interventions differed. The outcome was therefore downgraded one level for serious indirectness. Although only two studies with 182 participants were included, the 95% CI was narrow (−10.09 to −8.68), did not cross the null, and both limits suggested a marked between-group difference, so the outcome was not downgraded for imprecision; this did not offset serious limitations in other GRADE domains. Evidence came from only two single-center studies in China, and trial registries and gray literature were not searched; the outcome was downgraded one level for suspected publication bias. [d] HAMD was downgraded one level each for serious risk of bias and serious inconsistency (I^2^ = 81%). Evidence came from only two small studies with positive findings, and trial registries and gray literature were not searched; the outcome was downgraded one level for suspected publication bias. [e] Adverse events were incompletely reported in only two studies; most studies did not describe active monitoring procedures and very few events were observed. The evidence was therefore downgraded to very low certainty for serious risk of bias and very serious imprecision. Publication bias could not be excluded, but no further downgrading was applied because the evidence had already reached the lowest certainty category.

CI, confidence interval; GRADE, Grading of Recommendations Assessment, Development and Evaluation; HAMA, Hamilton Anxiety Rating Scale; HAMD, Hamilton Depression Rating Scale; MD, mean difference; OR, odds ratio; PSQI, Pittsburgh Sleep Quality Index.

### 2.8 Data synthesis

Meta-analyses were performed using RevMan 5.4.1. Dichotomous outcomes were pooled as odds ratios (ORs), and continuous outcomes as mean differences (MDs), each with 95% confidence intervals (CIs). Medians, interquartile ranges, or other statistics were not converted to means and standard deviations, and missing outcome data were not statistically imputed. Model selection considered the expected distribution of true effects across studies, clinical and methodological diversity, and statistical heterogeneity, rather than relying solely on the *p* value from the heterogeneity test or an I^2^ threshold. For trial-defined clinical response and HAMA scores, outcome measurement was sufficiently compatible, effects were directionally consistent, and no substantial statistical heterogeneity was observed; a fixed-effect model was therefore used for the primary analysis to estimate a common effect within the set of included studies. Because studies still differed in herbal composition, acupoint selection, treatment intensity, clinical setting, and co-interventions, DerSimonian–Laird random-effects models were additionally used for model-sensitivity analyses. Random-effects models were used as the primary analyses for other outcomes with appreciable clinical, methodological, or statistical heterogeneity. Heterogeneity was evaluated using the *p* value, I^2^ statistic, and between-study variance (τ^2^). I^2^ was interpreted cautiously when few studies were included; I^2^ = 0% was not considered proof that true between-study heterogeneity was absent. Leave-one-out sensitivity analyses sequentially excluded each study and reported the omitted study, pooled effect, 95% CI, *p* value, I^2^, and statistical model. Additional quantitative subgroup analyses by treatment duration, daily application time, acupoint selection, or herbal formula were considered, but were not performed because too few studies populated each category and one trial did not report treatment frequency, making such analyses unstable and potentially misleading.

## 3 Results

### 3.1 Study selection

**Figure 1:**
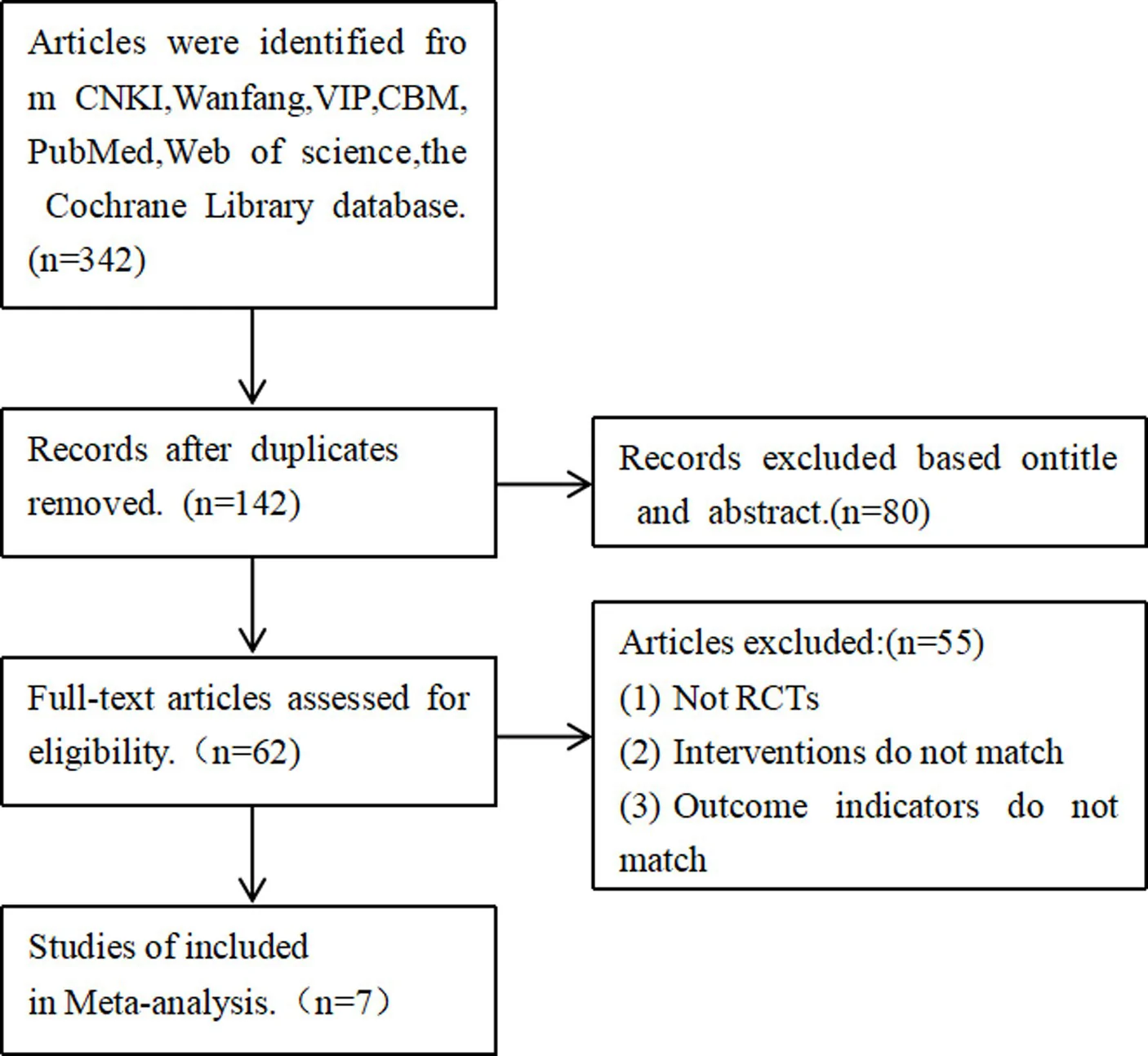
PRISMA flow diagram of study selection.

The search identified 342 records. After removal of duplicates and screening of titles, abstracts, and full texts, seven randomized controlled trials met the eligibility criteria. The study-selection process is shown in Figure 1.

### 3.2 Characteristics of included studies

The seven trials included 608 participants, with 304 in the experimental groups and 304 in the control groups (14–20). Studies were published between 2013 and 2025. Five studies reported trial-defined clinical response (15–19), six reported Pittsburgh Sleep Quality Index (PSQI) scores (14, 15, 17–20), two reported HAMA scores (19, 20), and two reported Hamilton Depression Rating Scale (HAMD) scores (19, 20). All studies added acupoint application to the comparator intervention. Comparators comprised conventional medical treatment alone or conventional medical treatment combined with acupuncture, rehabilitation training, graded psychological nursing, or an oral Chinese herbal formula. Treatment intensity varied markedly: daily application ranged from 0.5 to 24 h, treatment duration ranged from 10 days to 4 weeks, and one study did not report application frequency. No trial reported post-treatment follow-up. In the three-arm trial by Zhao et al. (19), only the comparison between the combined group (modified Chaihu Shugan Formula plus acupoint application) and the oral-treatment group (the same modified Chaihu Shugan Formula alone) was extracted to estimate the added effect of acupoint application; the external-application-only group was not included in this two-group comparison. Basic study characteristics are presented in Table 4, and additional clinical and reporting characteristics in Table 5.

**Table 4:** Basic characteristics of the included studies.

Study	Sample size (E/C)	Age (E/C), years	Sex (M/F)	Disease duration	Intervention	Frequency	Treatment duration	Outcomes
Shi Handan (17)	42 (26/16) / 42 (20/22)	68.72 ± 8.14/71.68 ± 7.17	E: 26/16; C: 20/22	NR	E: CWMT + RT + AA; C: CWMT + RT	4–6 h/day, 7 days/week	4 weeks	①②
Zhao Weidong (19)	50 (28/22) / 50 (26/24)	39.65 ± 4.24/40.06 ± 4.06	E: 28/22; C: 26/24	8.63 ± 2.16 months / 8.35 ± 2.10 months	E: CWMT + MCSGF + AA; C: CWMT + MCSGF	24 h/day, 7 days/week	2 weeks	①②③④
Yan Wei (18)	30 (14/16) / 30 (17/13)	73.90 ± 8.42/74.27 ± 7.41	E: 14/16; C: 17/13	3.17 ± 0.75 years / 3.12 ± 0.52 years	E: CWMT + AT + AA; C: CWMT + AT	NR	2 weeks	①②
Zhou Yiping (20)	41 (25/16) / 41 (23/18)	61.35 ± 4.71/61.42 ± 4.76	E: 25/16; C: 23/18	NR	E: CWMT + PC + AA; C: CWMT + PC	12 h/day, 7 days/week	4 weeks	②③④
Gao Shijuan (15)	75 (40/35) / 75 (42/33)	64.22 ± 8.07/65.47 ± 8.17	E: 40/35; C: 42/33	3.15 ± 1.07 weeks / 3.26 ± 1.24 weeks	E: CWMT + AT + AA; C: CWMT + AT	12 h/day, 6 days/week	2 weeks	①②
Chen Ying (14)	30 (15/15) / 30 (13/17)	59.57 ± 5.55/55.03 ± 9.88	E: 15/15; C: 13/17	NR	E: CWMT + AA; C: CWMT	2–4 h/day, 7 days/week	10 days	②
Jiang Meihua (16)	36 (21/15) / 36 (20/16)	64.6 ± 1.4/64.8 ± 1.5	E: 21/15; C: 20/16	10.5 ± 0.7 months / 10.7 ± 0.8 months	E: CWMT + AA; C: CWMT	0.5 h/day, 7 days/week	20 days	①

E, experimental group; C, control group; M, male; F, female; NR, not reported; CWMT, conventional Western medical treatment; RT, rehabilitation training; AT, acupuncture treatment; MCSGF, modified Chaihu Shugan Formula; PC, graded psychological nursing; AA, acupoint application. Outcomes: ① trial-defined clinical response; ② PSQI; ③ HAMD; ④ HAMA.

**Table 5:** Additional clinical and reporting characteristics of the included studies, including HAMA verification information.

Study	Stroke stage	Sleep-disorder diagnosis	Baseline severity and HAMA information	Herbal medicine/acupoints	Rationale for selection	Follow-up	Adverse-event monitoring
Shi Handan (17)	Post-stroke; specific stage NR	Post-stroke sleep disorder with heart–kidney non-interaction; insomnia lasting 4 weeks; formal diagnostic criteria NR	Baseline PSQI: E 15.61 ± 1.42; C 14.86 ± 1.17	Evodia rutaecarpa powder; Shenque (CV8), Neiguan (PC6), and Yongquan (KI1)	TCM theory of heart–kidney non-interaction	NR	NR
Zhao Weidong (19)	After cerebral infarction; specific stage NR	Met the cited Western medicine and TCM diagnostic criteria for insomnia; predominant pattern: liver constraint with yin deficiency	Baseline PSQI: E 13.01 ± 2.97; C 12.86 ± 3.26. Baseline HAMA: E 26.28 ± 4.08; C 25.79 ± 3.99. The HAMA had 14 items, each scored 0–4; higher scores indicated more severe anxiety. Endpoint: 2 weeks. Specific Chinese version, rater training, and inter-rater reliability NR.	Ningjing patch (composition NR); Neiguan (PC6) and Shenmen (HT7)	Liver constraint with yin deficiency; soothe the liver, relieve constraint, nourish blood, and calm the spirit	NR	Events reported; monitoring procedure NR
Yan Wei (18)	Post-stroke; specific stage NR	Post-stroke insomnia with heart–kidney non-interaction; Chinese Guidelines for the Diagnosis and Treatment of Adult Insomnia (2017); insomnia and syndrome criteria in Internal Medicine of Traditional Chinese Medicine; PSQI > 7	Baseline PSQI: E 16.83 ± 2.27; C 16.88 ± 2.65	Evodia rutaecarpa powder; Yongquan (KI1)	Pattern differentiation of heart–kidney non-interaction	NR	NR
Zhou Yiping (20)	Acute cerebral infarction; first-ever stroke; admitted < 6 h after onset	Sleep disorder; PSQI used to assess sleep quality	Baseline PSQI: E 11.82 ± 1.24; C 11.86 ± 1.25. Baseline HAMA: E 27.58 ± 4.08; C 27.65 ± 4.12. The HAMA had 14 items and higher scores indicated more severe anxiety. Endpoint: 4 weeks. Item-level scoring, specific Chinese version, rater training, and inter-rater reliability NR.	Cinnamon powder; Yongquan (KI1)	TCM meridian theory	NR	NR
Gao Shijuan (15)	Post-stroke; specific stage NR	Insomnia with yin deficiency and fire exuberance diagnosed according to the Criteria of Diagnosis and Therapeutic Effect of Diseases and Syndromes in Traditional Chinese Medicine	Baseline PSQI: E 13.63 ± 2.79; C 13.06 ± 2.40	Evodia rutaecarpa powder; Yongquan (KI1)	Theory of guiding fire back to its source and the pathogenesis of yin deficiency with fire exuberance	NR	Events reported; monitoring procedure NR
Chen Ying (14)	Stroke recovery stage	Insomnia diagnosed according to the Chinese Classification and Diagnostic Criteria of Mental Disorders; PSQI > 7	Baseline PSQI: E 15.63 ± 2.47; C 14.00 ± 2.35	Anshen powder (cinnamon powder, Evodia rutaecarpa powder, etc.); Shenmen (HT7)	The holistic concept of TCM and “external treatment for internal disease”	NR	NR
Jiang Meihua (16)	Post-stroke; specific stage NR	Western medicine diagnosis based on the Guiding Principles for Clinical Research on New Chinese Medicines; TCM diagnosis based on the Criteria of Diagnosis and Therapeutic Effect of Diseases and Syndromes in Traditional Chinese Medicine	NR	Huoxue powder (Angelica pubescens, Chuanxiong rhizome, and Chaenomeles fruit); Yongquan (KI1), Dazhui (GV14), Pishu (BL20), Zusanli (ST36), and Sanyinjiao (SP6)	TCM meridian theory and theories of the zang-fu organs, qi, and blood	NR	NR

AA, acupoint application; C, control group; E, experimental group; HAMA, Hamilton Anxiety Rating Scale; NR, not reported; PSQI, Pittsburgh Sleep Quality Index; TCM, traditional Chinese medicine.

### 3.3 Risk of bias

**Figure 2:**
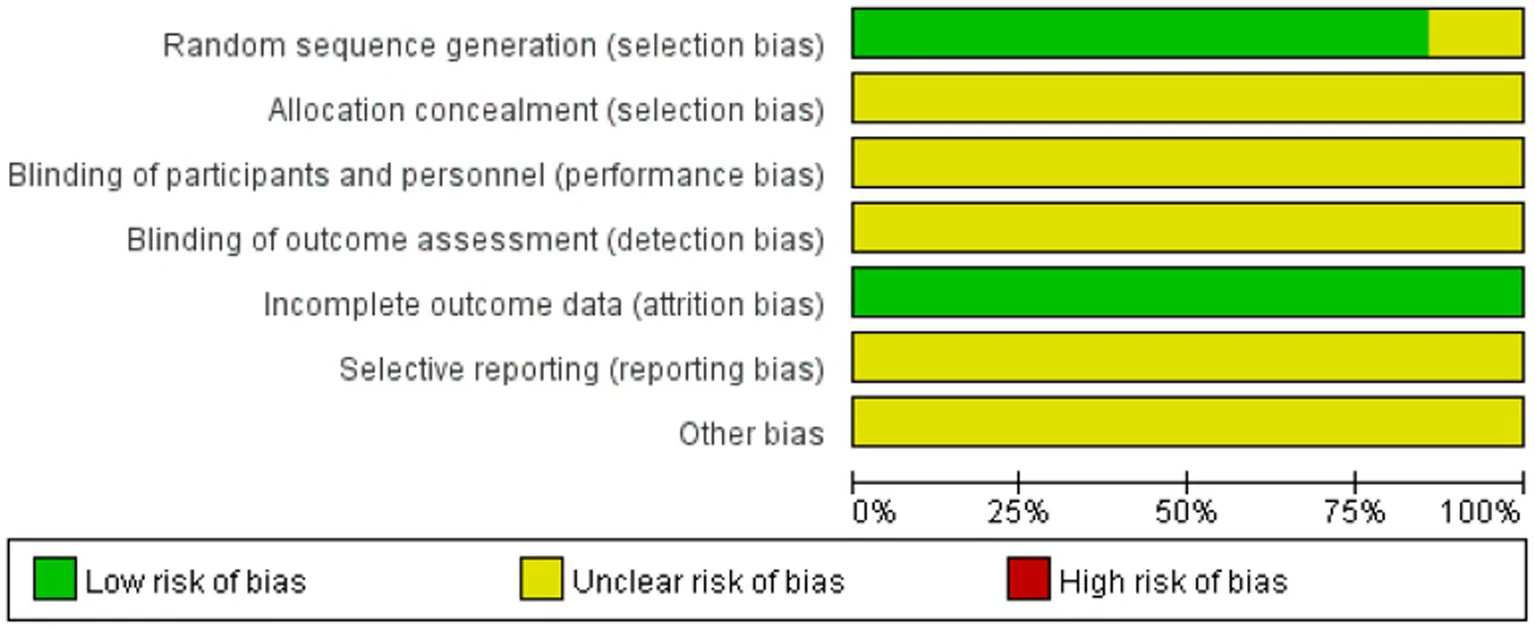
Risk-of-bias graph.

**Figure 3:**
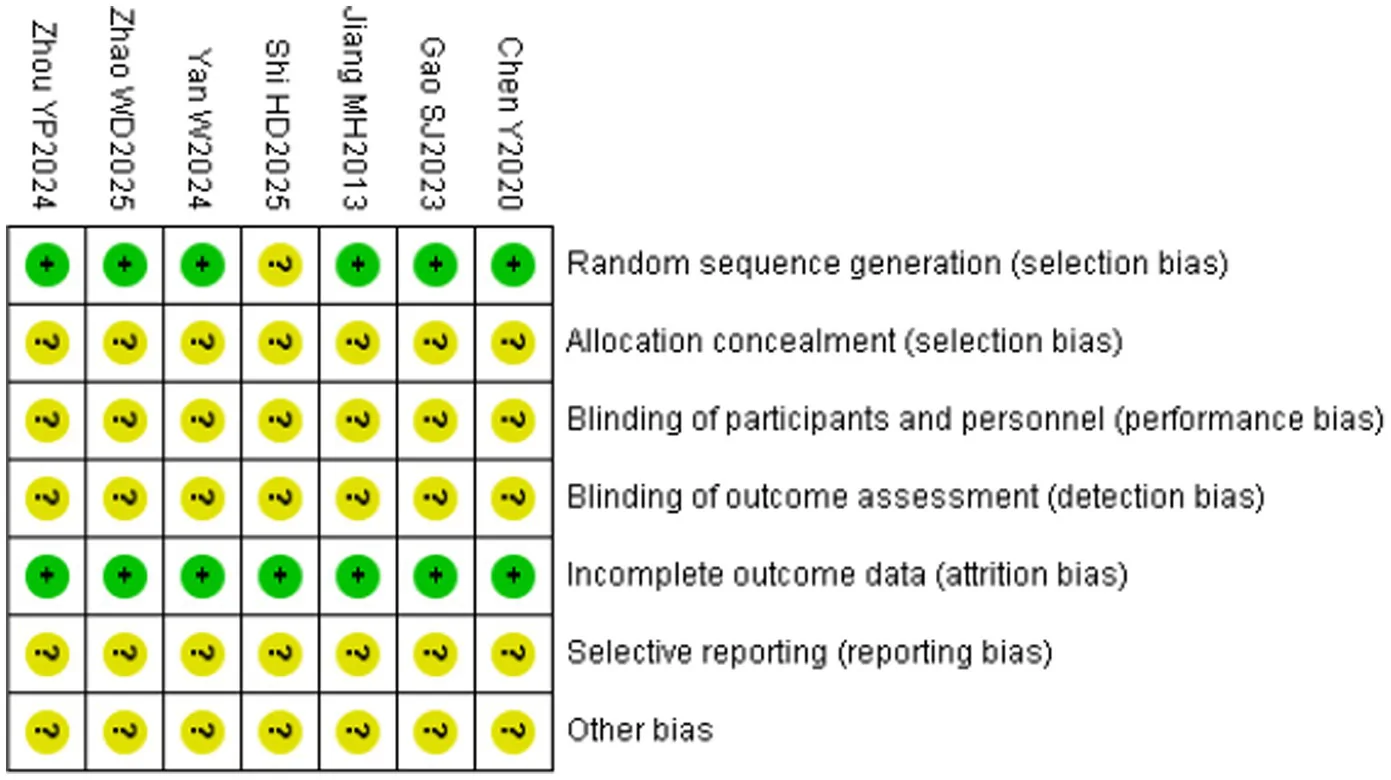
Risk-of-bias summary.

One study reported random allocation without describing how the random sequence was generated (17); the remaining studies described a randomization method (14–16, 18–20). No trial adequately reported allocation concealment, blinding of participants and personnel, or blinding of outcome assessors. No important missing outcome data were identified. No study protocol or trial-registration record was available for any included trial. Selective reporting was therefore judged to be at unclear risk in all trials: even when all outcomes listed in the published Methods section were reported, the possibility that other measured outcomes were omitted could not be excluded. Reassessment of the “other bias” domain identified no definite additional source of bias in any study. Nevertheless, the available reports were insufficient to determine whether design-specific problems, serious baseline imbalance, or other potential biases not captured by the standard domains were present; “other bias” was therefore rated unclear for all seven trials. Because PSQI is self-reported and HAMA and HAMD depend on assessor judgment, inadequate reporting of blinding for participants, personnel, and outcome assessors meant that performance and measurement bias could not be excluded and may have exaggerated the observed effects. Risk-of-bias judgments are shown in Figures 2, 3.

### 3.4 Meta-analysis

#### 3.4.1 Trial-defined clinical response

**Figure 4:**
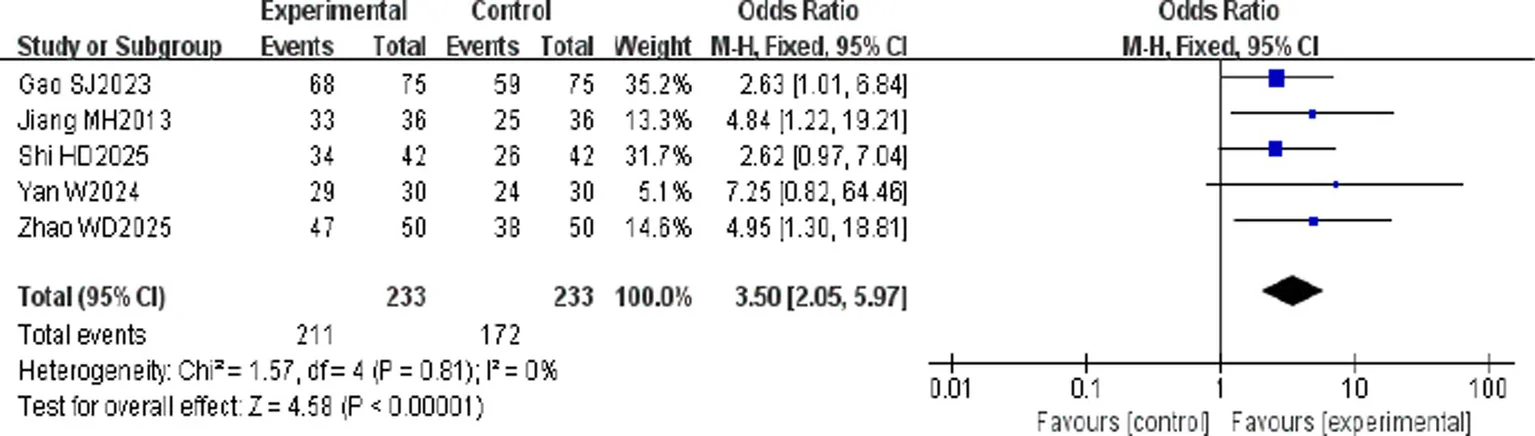
Forest plot of trial-defined clinical response.

Five studies involving 466 participants reported trial-defined clinical response (15–19). Statistical heterogeneity was low (*p* = 0.81, I^2^ = 0%). In the fixed-effect model, acupoint application plus the matched comparator intervention was associated with a higher trial-defined clinical response rate than the comparator intervention alone (OR = 3.50, 95% CI 2.05–5.97; Z = 4.58; *p* < 0.00001; Figure 4). Given persisting differences in acupoints, herbal formulas, treatment intensity, clinical settings, and co-interventions, a random-effects model-sensitivity analysis was also performed and yielded OR = 3.41 (95% CI 1.98–5.85; τ^2^ = 0.00; *p* < 0.00001). The effect direction, magnitude, and statistical significance were similar to those in the fixed-effect analysis. Because response definitions were not standardized, this pooled estimate should be interpreted as an aggregate of outcomes defined independently by each trial, not as a set of clinically equivalent response outcomes.

#### 3.4.2 Pittsburgh sleep quality index

**Figure 5:**
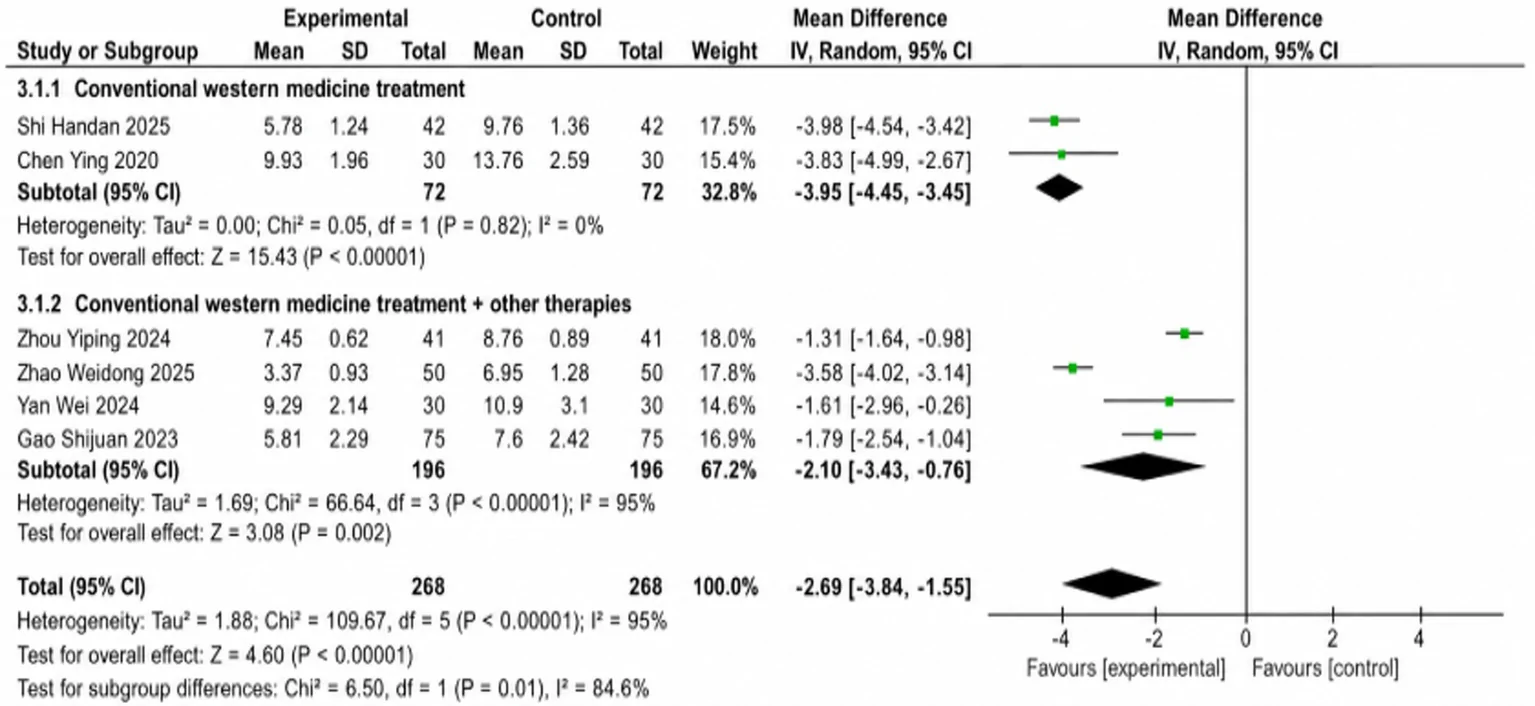
Forest plot of Pittsburgh Sleep Quality Index scores stratified by comparator intervention.

Six studies involving 536 participants reported PSQI scores (14, 15, 17–20). Heterogeneity was substantial (*p* < 0.00001, I^2^ = 95%). A random-effects model showed lower PSQI scores in the experimental groups (MD = −2.69, 95% CI − 3.84 to −1.55; Z = 4.60; *p* < 0.00001; Figure 5). In an exploratory subgroup analysis, the effect was larger when the comparator was conventional medical treatment alone (MD = −3.95, 95% CI − 4.45 to −3.45) and smaller when the comparator was conventional medical treatment plus another active intervention (MD = −2.10, 95% CI − 3.43 to −0.76). The between-subgroup difference was statistically significant (*p* = 0.01), but each subgroup contained few studies and the result should be interpreted cautiously. Further subgroup analyses by treatment duration, daily application time, acupoint selection, or herbal formula were considered unreliable because of the small number of studies and incomplete reporting.

#### 3.4.3 HAMA and HAMD scores

**Figure 6:**
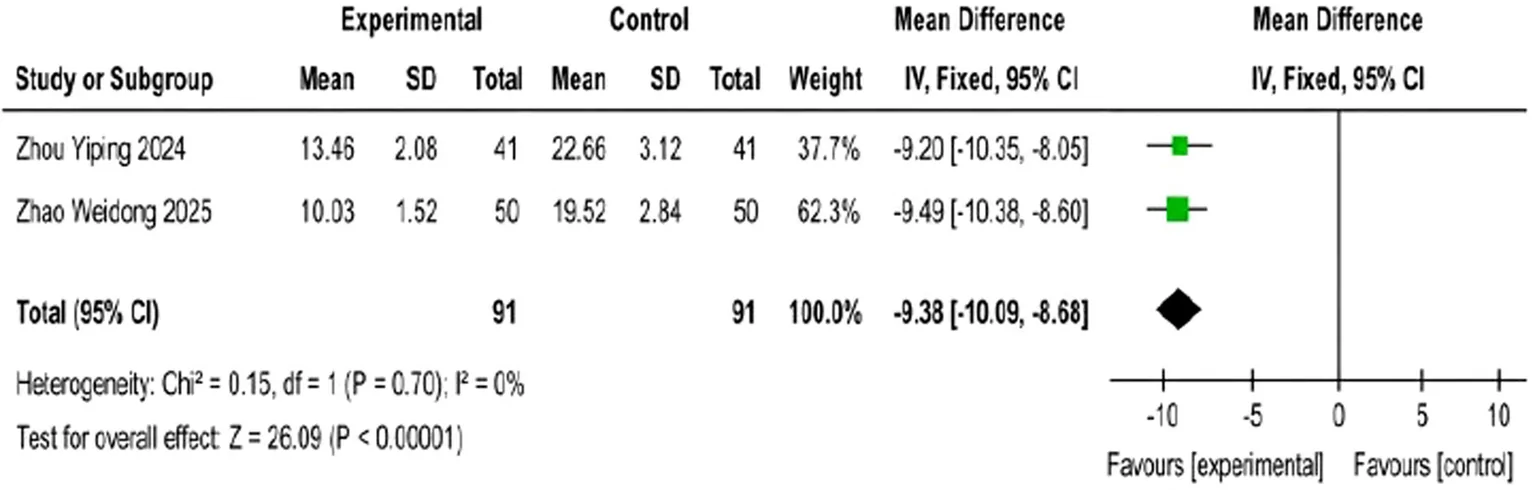
Forest plot of Hamilton Anxiety Rating Scale scores.

Two studies involving 182 participants reported HAMA scores (19, 20). After two reviewers rechecked the original full texts and data tables, the study by Zhou et al. (20) was confirmed to have assigned graded psychological nursing plus acupoint application to the experimental group and graded psychological nursing alone to the control group. After 4 weeks, mean HAMA scores were 13.46 ± 2.08 (*n* = 41) and 22.66 ± 3.12 (*n* = 41), respectively, corresponding to an MD of −9.20 (95% CI − 10.35 to −8.05). Zhao et al. (19) conducted a three-arm trial. For the prespecified added-effect comparison, the combined-treatment group was selected as the experimental group and the oral-treatment group as the control group, while the external-application-only group was excluded. After 2 weeks, mean HAMA scores were 10.03 ± 1.52 (*n* = 50) and 19.52 ± 2.84 (*n* = 50), respectively, corresponding to an MD of −9.49 (95% CI − 10.38 to −8.60). Means, standard deviations, sample sizes, group direction, and individual-study effects in the forest plot were consistent with the original reports. Statistical heterogeneity was low (*p* = 0.70, I^2^ = 0%), and the fixed-effect model showed lower HAMA scores in the experimental groups (MD = −9.38, 95% CI − 10.09 to −8.68; Z = 26.09; *p* < 0.00001; Figure 6). The random-effects model-sensitivity analysis gave the same estimate (MD = −9.38, 95% CI − 10.09 to −8.68; τ^2^ = 0.00; *p* < 0.00001).

**Figure 7:**
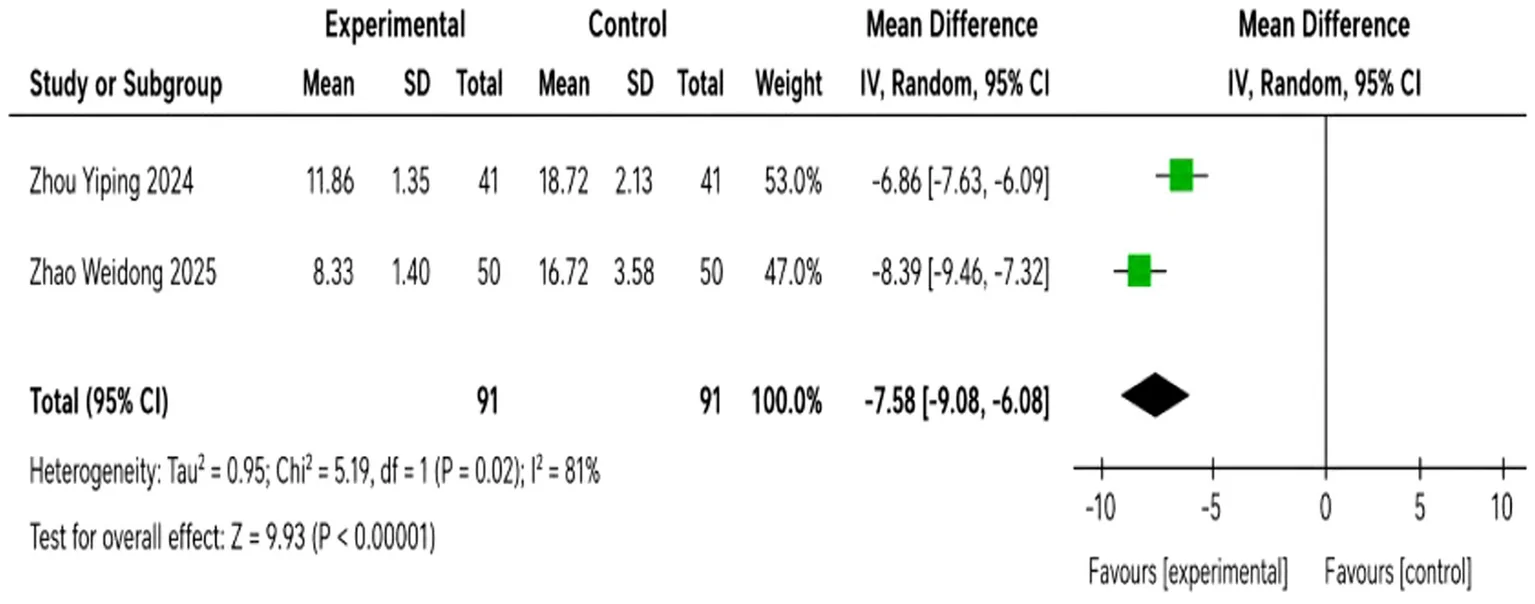
Forest plot of Hamilton Depression Rating Scale scores.

Both studies explicitly used the 14-item HAMA and indicated that higher scores represented more severe anxiety. Zhao et al. (19) further specified that each item was scored from 0 to 4, whereas Zhou et al. (20) did not report item-level scoring. The instruments were therefore reasonably comparable in item structure, scoring direction, and clinical interpretation, supporting use of the MD. However, neither study identified the specific Chinese translation or revision, and neither reported rater training or inter-rater reliability; use of exactly the same HAMA version could therefore not be confirmed. Only two studies contributed to this outcome, assessments occurred at 2 and 4 weeks, and the heterogeneity test had limited power; I^2^ = 0% could not exclude underlying between-study differences. The same two studies reported HAMD scores. Heterogeneity was substantial (*p* = 0.02, I^2^ = 81%), and a random-effects model showed lower HAMD scores in the experimental groups (MD = −7.58, 95% CI − 9.08 to −6.08; Z = 9.93; *p* < 0.00001; Figure 7).

#### 3.4.4 Safety

Only two studies reported adverse events (15, 19). One reported dry mouth, dizziness or fatigue, and skin allergy in the experimental group, and dry mouth, dizziness or fatigue, skin allergy, facial edema, and memory impairment in the control group (15). The other reported one case of nausea in the experimental group and one case of constipation in the control group (19). The remaining five studies did not state whether adverse events were actively monitored and did not explicitly report that no adverse events occurred. “Not reported” cannot be interpreted as “no adverse events”; the available evidence is insufficient to establish the safety of acupoint application.

### 3.5 Sensitivity analyses

In addition to leave-one-out analyses, model-sensitivity analyses were conducted for trial-defined clinical response and HAMA scores. Replacing the fixed-effect model with a random-effects model yielded OR = 3.41 (95% CI 1.98–5.85; τ^2^ = 0.00; *p* < 0.00001) for trial-defined clinical response and MD = −9.38 (95% CI − 10.09 to −8.68; τ^2^ = 0.00; *p* < 0.00001) for HAMA, with the same effect directions and statistical conclusions as the primary fixed-effect analyses. Leave-one-out sensitivity analyses were also performed for trial-defined clinical response and PSQI scores. Sequential omission of each study did not change the direction or statistical significance of the pooled effects, suggesting that no single trial drove the main findings. Trial-defined clinical response was reanalyzed five times using the fixed-effect model, and PSQI six times using the random-effects model. Each reanalysis recorded the omitted study, pooled effect, 95% CI, *p* value, I^2^, and model; complete results are provided in Supplementary Table S1. These analyses support the statistical robustness of the findings but do not remove the underlying clinical and methodological heterogeneity.

### 3.6 Certainty of evidence

GRADE assessments indicated low-certainty evidence for trial-defined clinical response and very-low-certainty evidence for PSQI, HAMA, HAMD, and adverse events. Evidence for trial-defined clinical response was downgraded for serious risk of bias and suspected publication bias. Although background treatments or co-interventions varied across studies—including conventional treatment, acupuncture, rehabilitation, psychological nursing, and oral Chinese herbal medicine—each trial compared acupoint application plus a background intervention with the same background intervention alone, so co-interventions were matched within each trial. Differences in co-interventions primarily limited generalizability across clinical settings but were not judged sufficient to constitute serious indirectness, and the outcome was not additionally downgraded for indirectness. Trial-defined response criteria also differed, so the pooled estimate represents an aggregate of outcomes as defined by individual trials. PSQI evidence was downgraded for serious risk of bias, serious inconsistency, and suspected publication bias. HAMA evidence was downgraded for serious risk of bias; serious indirectness arising from unreported Chinese translations or revisions, rater training, and inter-rater reliability, as well as different assessment times and heterogeneous co-interventions; and suspected publication bias. Although only two studies with 182 participants contributed to HAMA, the pooled 95% CI was narrow (−10.09 to −8.68), did not cross the null, and both limits suggested a marked between-group difference; the outcome was therefore not downgraded for imprecision. This statistical precision did not offset uncertainty caused by risk of bias, indirectness, and suspected publication bias. HAMD evidence was downgraded for serious risk of bias, serious inconsistency, and suspected publication bias. Trial-defined clinical response, PSQI, HAMA, and HAMD were each downgraded one level for suspected publication bias because the evidence bases were small, all studies were published in Chinese and most were small single-center trials, results generally favored the intervention group, trial registries and gray literature were not searched, and these were excluded according to the prespecified protocol. Unpublished studies, negative findings, and selective publication could therefore not be excluded. Fewer than 10 studies contributed to every outcome, so formal funnel-plot or regression testing was not performed; inability to conduct a formal test does not imply absence of publication bias. Adverse-event evidence was downgraded to very low certainty because monitoring and reporting were incomplete and events were very few. Publication bias could not be excluded, but the evidence was not downgraded further because it had already reached the lowest GRADE category, not because publication bias was considered absent. Outcome-specific downgrading judgments are detailed in the footnotes to Table 3.

## 4 Discussion

### 4.1 Principal findings

This systematic review included seven randomized controlled trials with 608 participants. Pooled analyses suggest that adding acupoint application to conventional treatment may increase trial-defined clinical response and reduce PSQI, HAMA, and HAMD scores. However, the GRADE certainty ratings were low to very low, indicating limited confidence in the effect estimates and a substantial possibility that the true effects differ from the observed results. Evidence on adverse events was of very low certainty and did not support a definitive safety conclusion. Post-stroke sleep disorders may adversely affect participation in rehabilitation, emotional regulation, cognitive recovery, and quality of life (21–23). Fixed-effect primary analyses and random-effects model-sensitivity analyses for trial-defined clinical response and HAMA were consistent, indicating some statistical robustness to model assumptions; this agreement should not be interpreted as complete clinical or methodological homogeneity among studies.

### 4.2 Sleep outcomes and heterogeneity

PSQI was the most direct sleep-related outcome and covers subjective sleep quality, sleep latency, sleep duration, sleep efficiency, sleep disturbances, use of sleep medication, and daytime dysfunction; lower scores indicate better perceived sleep. Recent post-stroke sleep studies have also used PSQI-based outcomes to describe poor sleep quality and insomnia symptoms (22, 24). However, heterogeneity in the PSQI analysis was very high (I^2^ = 95%). Studies differed in daily application time (0.5–24 h), weekly frequency, treatment duration (10 days to 4 weeks), acupoint selection, herbal composition, stroke stage, baseline sleep severity, rehabilitation setting, and background treatment, reflecting substantial variation in treatment intensity and clinical context. Unreported differences in stroke severity, rehabilitation phase, and sleep-disorder phenotype may have contributed additional between-study variation. The exploratory subgroup analysis suggested that comparator intensity might explain part of the heterogeneity, but the number of studies in each subgroup was small and no firm conclusion can be drawn. Because studies were few and reporting incomplete, further subgroup analyses by treatment intensity, acupoint, or herbal formula could not be performed reliably.

### 4.3 Emotional outcomes and risk of bias

The pooled findings also suggest possible improvement in anxiety and depressive symptoms. Post-stroke sleep disorders, anxiety, and depression have bidirectional relationships: anxiety may increase nocturnal awakenings and prolong sleep latency; depression may worsen early awakening, sleep fragmentation, and daytime fatigue; and persistent poor sleep may impair emotional regulation and reduce confidence in rehabilitation. These relationships are consistent with recent evidence linking post-stroke sleep disorders to depression, anxiety, and cognitive outcomes (25–27). Rechecking the two original reports identified no extraction error in HAMA means, standard deviations, sample sizes, group assignments, or assessment times. The individual MDs were −9.20 and −9.49, with small within-group standard deviations, producing an unusually large pooled effect and narrow CI; this statistical precision does not imply high certainty of evidence. Both studies used the 14-item HAMA with the same scoring direction and were comparable in item structure and clinical interpretation, but neither reported the specific Chinese translation or revision, rater training, or inter-rater reliability. Assessments were conducted after 2 and 4 weeks, and the background co-interventions differed. More importantly, neither study described allocation concealment or blinding of outcome assessors. Because HAMA and HAMD depend on assessor judgment, the observed effects are particularly vulnerable to performance, measurement, and selective-reporting biases and may have been overestimated.

### 4.4 Theoretical considerations and clinical implications

In traditional Chinese medicine theory, post-stroke sleep disorders are commonly attributed to disharmony of qi and blood, imbalance of yin and yang, obstruction of the collaterals by phlegm and stasis, and inadequate nourishment of the spirit. Acupoints frequently selected in the included trials were Yongquan (KI1), Shenmen (HT7), Shenque (CV8), Neiguan (PC6), Sanyinjiao (SP6), and Zusanli (ST36). Proposed modern biomedical explanations include local sensory stimulation, transdermal absorption of herbal constituents, autonomic regulation, improved microcirculation, and neuroendocrine–immune modulation. These mechanisms remain hypothetical: none of the included trials measured mechanism-related biomarkers or directly tested causal pathways, and the clinical evidence cannot establish a mechanism of action. In nursing and rehabilitation practice, acupoint application should be considered, at most, an adjunct within comprehensive care and not a substitute for standard post-stroke treatment. Its noninvasive nature may facilitate combination with sleep-hygiene education, psychological support, medication management, and rehabilitation. This positioning is consistent with evidence that nonpharmacological, noninvasive interventions may have a role in post-stroke sleep management, although efficacy and implementation conditions require cautious evaluation (23, 28).

### 4.5 Safety, follow-up, and reporting completeness

Safety cannot be inferred from a lack of reporting. Only two studies described adverse events, the other five did not state whether active monitoring was performed, and no study reported a detailed monitoring procedure. Future clinical applications and trials should prospectively define and systematically record local skin reactions, systemic symptoms, contraindications, application duration, and adherence. Current evidence reflects only short-term outcomes at the end of treatment. No trial reported post-treatment follow-up, and treatment duration ranged from 10 days to 4 weeks. It therefore remains unclear whether sleep or emotional benefits persist, delayed skin reactions occur, symptoms recur, or the intervention affects long-term neurological recovery or quality of life. The absence of follow-up substantially limits clinical applicability. The expanded characteristics table also shows that clinically important information was frequently missing. Only a few studies clearly specified stroke stage. Most reported some diagnostic basis for the sleep disorder, baseline PSQI, and a rationale for treatment selection, but the complete herbal composition remained unclear in some studies, and none described a specific adverse-event monitoring plan. Recording missing information as NR improves transparency but cannot compensate for poor reporting in the primary studies.

### 4.6 Strengths and limitations

This systematic review used a prospectively registered protocol, followed PRISMA 2020, used two independent reviewers for study selection and data extraction, assessed risk of bias with RoB 1, performed leave-one-out and model-sensitivity analyses, and evaluated certainty using GRADE. Important limitations remain. First, all included studies were published in Chinese and most appeared to be small, single-center trials, limiting generalizability and raising concern about publication bias. Second, no trial clearly reported allocation concealment or blinding. Because protocols, registration records, and sufficient design details were unavailable, selective reporting and other bias were rated unclear. The main outcomes were subjective or semi-subjective and treatment effects may have been overestimated. Third, marked differences in treatment intensity, acupoints, herbal preparations, co-interventions, trial-defined response criteria, and clinical context limited interpretation of pooled estimates. Although I^2^ was 0% for trial-defined clinical response and HAMA, these outcomes included only five and two studies, respectively, and heterogeneity tests had limited power; potential differences in true effects cannot be excluded. For HAMA in particular, assessments occurred at 2 and 4 weeks, co-interventions differed, and the specific Chinese translation, rater training, and inter-rater reliability were not reported. Agreement between fixed-effect and random-effects analyses supports some statistical robustness to model assumptions but does not eliminate clinical and methodological diversity. Fourth, trial registries and gray literature were not systematically searched, and master’s and doctoral theses were excluded according to the prespecified protocol; unpublished or negative findings may therefore have been missed. Fifth, objective sleep measures and post-treatment follow-up were absent. Finally, adverse-event reporting was sparse and could not support a safety conclusion.

### 4.7 Future research and conclusion

Future trials should use multicenter randomized designs, ensure adequate allocation concealment, blind outcome assessors where feasible, prospectively register protocols, and report transparently and completely. Standardized acupoint-application protocols should specify acupoints, complete herbal composition, preparation method, dose, daily application time, treatment duration, skin preparation, adherence, contraindications, and adverse-event monitoring. Trials should prespecify clinically interpretable response definitions and report event counts by group. For assessor-rated scales such as HAMA and HAMD, investigators should identify the specific Chinese version, number of items, item-level scoring range, scoring direction, assessment time, rater training, assessor blinding, and inter-rater reliability. Future research should combine validated subjective instruments with objective sleep outcomes such as actigraphy or polysomnography, include clinically meaningful follow-up, and evaluate neurological function, cognition, activities of daily living, adherence to rehabilitation, and quality of life. In conclusion, low- to very-low-certainty evidence suggests that Chinese herbal acupoint application as an adjunctive intervention may improve subjective sleep quality and emotional symptoms after stroke, but these findings remain preliminary. Safety has not been established and long-term effectiveness is unknown. Acupoint application should not be regarded as an established treatment for post-stroke sleep management until larger, rigorously designed, transparently reported trials with systematic safety monitoring and longer follow-up are available.

## Data Availability

The original contributions presented in the study are included in the article/Supplementary material, further inquiries can be directed to the corresponding author.
