# Supplementary material for: Efficacy and safety of Chinese herbal acupoint application for post-stroke sleep disorders: a systematic review and meta-analysis
Source: Front Neurol. 2026 Sep 10;17:1881026. doi: 10.3389/fneur.2026.1881026 (PMC13604025; doi:10.3389/fneur.2026.1881026)
Supplement: Supplementary file 1 [file Supplementary_file_1.docx]

**中文数据库检索（Chinese Database Search）**

**1相关主题词、同义词及近义词（Related keywords, synonyms, and near-synonyms）**

**1.1脑卒中（stroke）**

| 主题词 | 卒中 |
| --- | --- |
| 同义词及近义词 | 急性卒中、脑卒中、脑中风、脑血管意外、CVA、 CVAs、脑血管中风、中风、急性、急性脑血管意外、急性脑卒中、缺血性卒中、出血性卒中、脑梗死、卒中后、缺血性脑卒中、急性缺血性脑卒中、脑血管病、脑梗塞、出血性脑卒中 |

**1.2睡眠障碍（dyssomnia）**

| 主题词 | 睡眠障碍 |
| --- | --- |
| 同义词及近义词 | 多梦、梦交、梦行症、失眠症、睡眠过多、睡眠质量、睡眠、失眠、入睡和睡眠障碍、不寐 |

**1.3穴位贴敷（Acupoint Patch Therapy）**

| 主题词 | 无 |
| --- | --- |
| 同义词及近义词 | 穴位贴敷疗法、穴位贴敷法、三伏贴、中药贴敷、中药穴位贴敷、穴位贴敷疗法、穴位贴敷、穴位敷贴、中药穴位敷贴、贴敷、敷贴、伏九贴 |

**2检索过程及检索式（Retrieval Process and Retrieval Query）**

**2.1知网（CNKI）**

| 检索方式 | 高级检索 |
| --- | --- |
| 检索字段 | 篇关摘 |
| 检索时段 | 建库-至今（2026-04-01） |
| 篇关摘 | 卒中 + 急性卒中 + 脑卒中 + 脑中风 + 脑血管意外 + "CVA" + "CVAs" + 中风 + 脑血管中风 + 急性脑血管意外 |
| OR篇关摘 | 急性脑卒中 + 缺血性卒中 + 出血性卒中 + 脑梗死 + 卒中后 + 缺血性脑卒中 + 急性缺血性脑卒中 + 脑血管病 + 脑梗塞 + 出血性脑卒中 |
| AND篇关摘 | 睡眠障碍 + 多梦 + 梦交 + 梦行症 + 失眠症 + 睡眠过多 + 睡眠质量 + 睡眠 + 失眠 + 入睡和睡眠障碍 + 不寐 |
| AND篇关摘 | 穴位贴敷疗法 + 穴位贴敷法 + 三伏贴 + 中药贴敷 + 中药穴位贴敷 + 穴位贴敷疗法 + 穴位贴敷 + 穴位敷贴 + 中药穴位敷贴 + 贴敷 + 敷贴 + 伏九贴 |

检索式截图：（Search query screenshot）

检索过程截图（Search process screenshot）

**2.2万方**（Wanfang Data）

| 检索方式 | 高级检索 |
| --- | --- |
| 检索字段 | 主题 |
| 检索时段 | 建库-至今（2026-04-01） |
| 主题 | 卒中 OR 急性卒中 OR 脑卒中 OR 脑中风 OR 脑血管意外 OR "CVA" OR "CVAs" OR 中风 OR 脑血管中风 OR 急性脑血管意外 OR 急性脑卒中 OR 缺血性卒中 OR 出血性卒中 OR 脑梗死 OR 卒中后 OR 缺血性脑卒中 OR 急性缺血性脑卒中 OR 脑血管病 OR 脑梗塞 OR 出血性脑卒中 |
| AND主题 | 睡眠障碍 OR 多梦 OR 梦交 OR 梦行症 OR 失眠症 OR 睡眠过多 OR 睡眠质量 OR 睡眠 OR 失眠 OR 入睡和睡眠障碍 OR 不寐 |
| AND主题 | 穴位贴敷疗法 OR 穴位贴敷法 OR 三伏贴 OR 中药贴敷 OR 中药穴位贴敷 OR 穴位贴敷疗法 OR 穴位贴敷 OR 穴位敷贴 OR 中药穴位敷贴 OR 贴敷 OR 敷贴 OR 伏九贴 |

检索式截图：（Search query screenshot）

检索过程截图（Search process screenshot）

**2.3维普**（VIP Database）

| 检索方式 | 高级检索 |
| --- | --- |
| 检索字段 | 题名或关键词 |
| 检索时段 | 建库-至今（2026-04-01） |
| 题名或关键词 | 卒中 OR 急性卒中 OR 脑卒中 OR 脑中风 OR 脑血管意外 OR "CVA" OR "CVAs" OR 中风 OR 脑血管中风 OR 急性脑血管意外 OR 急性脑卒中 OR 缺血性卒中 OR 出血性卒中 OR 脑梗死 OR 卒中后 OR 缺血性脑卒中 OR 急性缺血性脑卒中 OR 脑血管病 OR 脑梗塞 OR 出血性脑卒中 |
| AND题名或关键词 | 睡眠障碍 OR 多梦 OR 梦交 OR 梦行症 OR 失眠症 OR 睡眠过多 OR 睡眠质量 OR 睡眠 OR 失眠 OR 入睡和睡眠障碍 OR 不寐 |
| AND题名或关键词 | 穴位贴敷疗法 OR 穴位贴敷法 OR 三伏贴 OR 中药贴敷 OR 中药穴位贴敷 OR 穴位贴敷疗法 OR 穴位贴敷 OR 穴位敷贴 OR 中药穴位敷贴 OR 贴敷 OR 敷贴 OR 伏九贴 |

检索式截图（Search query screenshot）

检索过程截图（Search process screenshot）

**2.4中国生物医学文献服务系统（SinoMed）**

| 检索方式 | 高级检索 |
| --- | --- |
| 检索字段 | 常用字段 |
| 检索时段 | 建库-至今（2026-04-01） |
| 常用字段 | 卒中 OR 急性卒中 OR 脑卒中 OR 脑中风 OR 脑血管意外 OR "CVA" OR "CVAs" OR 中风 OR 脑血管中风 OR 急性脑血管意外 OR 急性脑卒中 OR 缺血性卒中 OR 出血性卒中 OR 脑梗死 OR 卒中后 OR 缺血性脑卒中 OR 急性缺血性脑卒中 OR 脑血管病 OR 脑梗塞 OR 出血性脑卒中 |
| AND常用字段 | 睡眠障碍 OR 多梦 OR 梦交 OR 梦行症 OR 失眠症 OR 睡眠过多 OR 睡眠质量 OR 睡眠 OR 失眠 OR 入睡和睡眠障碍 OR 不寐 |
| AND常用字段 | 穴位贴敷疗法 OR 穴位贴敷法 OR 三伏贴 OR 中药贴敷 OR 中药穴位贴敷 OR 穴位贴敷疗法 OR 穴位贴敷 OR 穴位敷贴 OR 中药穴位敷贴 OR 贴敷 OR 敷贴 OR 伏九贴 |

**英文数据库检索（English Database Search）**

**1相关主题词、自由词（Related subject terms, free terms）**

**1.1脑卒中（stroke）**

| 数据库 | 主题词（查对应词表） |
| --- | --- |
| PubMed | Stroke |
| Cochrane Library | Stroke |
| Embase | cerebrovascular accident |

**汇总相关自由词（Aggregate relevant free terms）**

"Strokes" OR "Cerebrovascular Accidents" OR "Cerebral Stroke" OR "Cerebral Strokes" OR "Stroke, Cerebral" OR "Strokes, Cerebral" OR "Cerebrovascular Apoplexy" OR "Apoplexy, Cerebrovascular" OR "Vascular Accident, Brain" OR "Brain Vascular Accident" OR "Brain Vascular Accidents" OR "Vascular Accidents, Brain" OR "Cerebrovascular Stroke" OR "Cerebrovascular Strokes" OR "Stroke, Cerebrovascular" OR "Strokes, Cerebrovascular" OR "Apoplexy" OR "CVA" OR "CVAs" OR "Stroke, Acute" OR "Acute Stroke" OR "Acute Strokes" OR "Strokes, Acute" OR "Cerebrovascular Accident, Acute" OR "Acute Cerebrovascular Accident" OR "Acute Cerebrovascular Accidents" OR "Cerebrovascular Accidents, Acute" OR "Brain Infarction" OR "Brain Stem Infarctions +" OR "Cerebral Infarction +" OR "Hemorrhagic Stroke" OR "Ischemic Stroke" OR "Embolic Stroke" OR "Thrombotic Stroke +" OR "accident, cerebrovascular" OR "acute cerebrovascular lesion" OR "acute focal cerebral vasculopathy" OR "apoplectic stroke" OR "apoplexia" OR "blood flow disturbance, brain" OR "brain accident" OR "brain attack" OR "brain blood flow disturbance" OR "brain insult" OR "brain insultus" OR "cerebral apoplexia" OR "cerebral insult" OR "cerebral vascular accident" OR "cerebral vascular insufficiency" OR "cerebro vascular accident" OR "cerebrovascular arrest" OR "cerebrovascular failure" OR "cerebrovascular injury" OR "cerebrovascular insufficiency" OR "cerebrovascular insult" OR "cerebrum vascular accident" OR "cryptogenic stroke" OR "insultus cerebralis" OR "ischaemic seizure" OR "ischemic seizure" OR "cerebrovascular accident"

**1.2睡眠障碍（dyssomnia）**

| 数据库 | 主题词（查对应词表） |
| --- | --- |
| PubMed | Sleep Wake Disorders |
| Cochrane Library | Sleep Wake Disorders |
| Embase | sleep disorder |

**汇总相关自由词（Aggregate relevant free terms）**

"Disorder, Sleep Wake" OR "Disorders, Sleep Wake" OR "Sleep Wake Disorder" OR "Wake Disorder, Sleep" OR "Wake Disorders, Sleep" OR "Sleep Disorders" OR "Disorder, Sleep" OR "Disorders, Sleep" OR "Long Sleeper Syndrome" OR "Long Sleeper Syndromes" OR "Sleeper Syndrome, Long" OR "Sleeper Syndromes, Long" OR "Syndrome, Long Sleeper" OR "Syndromes, Long Sleeper" OR "Short Sleeper Syndrome" OR "Short Sleeper Syndromes" OR "Sleeper Syndrome, Short" OR "Sleeper Syndromes, Short" OR "Syndrome, Short Sleeper" OR "Syndromes, Short Sleeper" OR "Short Sleep Phenotype" OR "Phenotype, Short Sleep" OR "Phenotypes, Short Sleep" OR "Short Sleep Phenotypes" OR "Sleep Phenotypes, Short" OR "Sleep-Related Neurogenic Tachypnea" OR "Neurogenic Tachypnea, Sleep-Related" OR "Neurogenic Tachypneas, Sleep-Related" OR "Sleep Related Neurogenic Tachypnea" OR "Sleep-Related Neurogenic Tachypneas" OR "Tachypnea, Sleep-Related Neurogenic" OR "Tachypneas, Sleep-Related Neurogenic" OR "Subwakefullness Syndrome" OR "Subwakefullness Syndromes" OR "Syndromes, Subwakefullness" OR "Syndrome, Subwakefullness" OR "Dyssomnias" OR "Sleep Deprivation" OR "Sleep Disorders, Circadian Rhythm +" OR "Sleep Disorders, Intrinsic +" OR "Parasomnias" OR "Nocturnal Myoclonus Syndrome" OR "Nocturnal Paroxysmal Dystonia" OR "REM Sleep Parasomnias +" OR "Restless Legs Syndrome" OR "Sleep Arousal Disorders +" OR "Sleep Bruxism" OR "Sleep-Wake Transition Disorders" OR "atypical sleep" OR "difficult sleeping" OR "difficulties in sleeping" OR "difficulty sleeping" OR "disorder during sleep" OR "disorders during sleep" OR "disturbance during sleep" OR "disturbance of sleep" OR "disturbances during sleep" OR "disturbances of sleep" OR "problem during sleep" OR "problem of sleep" OR "problems during sleep" OR "problems of sleep" OR "sleep disturbance" OR "sleep disturbances" OR "sleep interference" OR "sleep perturbation" OR "sleep perturbations" OR "sleep problem" OR "sleep problems" OR "sleep related disease" OR "sleep related disorder" OR "sleep related problem" OR "sleeping difficulties" OR "sleeping difficulty" OR "sleeping disorder" OR "sleeping disorders" OR "sleeping problem" OR "sleeping problems" OR "trouble sleeping"

**1.3穴位贴敷（Acupoint Patch Therapy）**

| 数据库 | 主题词（查对应词表） |
| --- | --- |
| PubMed | 无 |
| Cochrane Library | 无 |
| Embase | 无 |

**汇总相关自由词（Aggregate relevant free terms）**

"acupoint herbal patching" OR "acupoint application" OR "acupoint herbal plaster" OR "acupoint patch" OR "acupoint patching" OR "acupoint sticking" OR "point application" OR "point application therapy" OR "acupoint herbal patching" OR "AHP" OR "Chinese acupoint application" OR "herbal application of acupoint paste" OR "Xueweitiefu" OR "acupressure application" OR "Drug Acupoint Application" OR "Herbal Patch" OR "Acupoint Patch" OR "External Application Therapy" OR "Plaster Therapy" OR "acupoint plaster" OR "herbal patching"

**2检索过程及检索式（Retrieval Process and Retrieval Query）**

**2.1 PubMed**

| 检索方式 | Advanced |
| --- | --- |
| 检索字段 | MeSH Terms、Title/Abstract |
| 检索时段 | 建库-至今（2026-04-01） |
| #1 |  |
| MeSH Terms | Stroke |
| OR Title/Abstract | "cerebrovascular accident" OR "Strokes" OR "Cerebrovascular Accidents" OR "Cerebral Stroke" OR "Cerebral Strokes" OR "Stroke, Cerebral" OR "Strokes, Cerebral" OR "Cerebrovascular Apoplexy" OR "Apoplexy, Cerebrovascular" OR "Vascular Accident, Brain" OR "Brain Vascular Accident" OR "Brain Vascular Accidents" OR "Vascular Accidents, Brain" OR "Cerebrovascular Stroke" OR "Cerebrovascular Strokes" OR "Stroke, Cerebrovascular" OR "Strokes, Cerebrovascular" OR "Apoplexy" OR "CVA" OR "CVAs" OR "Stroke, Acute" OR "Acute Stroke" OR "Acute Strokes" OR "Strokes, Acute" OR "Cerebrovascular Accident, Acute" OR "Acute Cerebrovascular Accident" OR "Acute Cerebrovascular Accidents" OR "Cerebrovascular Accidents, Acute" OR "Brain Infarction" OR "Brain Stem Infarctions +" OR "Cerebral Infarction +" OR "Hemorrhagic Stroke" OR "Ischemic Stroke" OR "Embolic Stroke" OR "Thrombotic Stroke +" OR "accident, cerebrovascular" OR "acute cerebrovascular lesion" OR "acute focal cerebral vasculopathy" OR "apoplectic stroke" OR "apoplexia" OR "blood flow disturbance, brain" OR "brain accident" OR "brain attack" OR "brain blood flow disturbance" OR "brain insult" OR "brain insultus" OR "cerebral apoplexia" OR "cerebral insult" OR "cerebral vascular accident" OR "cerebral vascular insufficiency" OR "cerebro vascular accident" OR "cerebrovascular arrest" OR "cerebrovascular failure" OR "cerebrovascular injury" OR "cerebrovascular insufficiency" OR "cerebrovascular insult" OR "cerebrum vascular accident" OR "cryptogenic stroke" OR "insultus cerebralis" OR "ischaemic seizure" OR "ischemic seizure" OR "cerebrovascular accident" |
| #2 |  |
| MeSH Terms | "Sleep Wake Disorders" |
| OR Title/Abstract | "sleep disorder" OR "Disorder, Sleep Wake" OR "Disorders, Sleep Wake" OR "Sleep Wake Disorder" OR "Wake Disorder, Sleep" OR "Wake Disorders, Sleep" OR "Sleep Disorders" OR "Disorder, Sleep" OR "Disorders, Sleep" OR "Long Sleeper Syndrome" OR "Long Sleeper Syndromes" OR "Sleeper Syndrome, Long" OR "Sleeper Syndromes, Long" OR "Syndrome, Long Sleeper" OR "Syndromes, Long Sleeper" OR "Short Sleeper Syndrome" OR "Short Sleeper Syndromes" OR "Sleeper Syndrome, Short" OR "Sleeper Syndromes, Short" OR "Syndrome, Short Sleeper" OR "Syndromes, Short Sleeper" OR "Short Sleep Phenotype" OR "Phenotype, Short Sleep" OR "Phenotypes, Short Sleep" OR "Short Sleep Phenotypes" OR "Sleep Phenotypes, Short" OR "Sleep-Related Neurogenic Tachypnea" OR "Neurogenic Tachypnea, Sleep-Related" OR "Neurogenic Tachypneas, Sleep-Related" OR "Sleep Related Neurogenic Tachypnea" OR "Sleep-Related Neurogenic Tachypneas" OR "Tachypnea, Sleep-Related Neurogenic" OR "Tachypneas, Sleep-Related Neurogenic" OR "Subwakefullness Syndrome" OR "Subwakefullness Syndromes" OR "Syndromes, Subwakefullness" OR "Syndrome, Subwakefullness" OR "Dyssomnias" OR "Sleep Deprivation" OR "Sleep Disorders, Circadian Rhythm +" OR "Sleep Disorders, Intrinsic +" OR "Parasomnias" OR "Nocturnal Myoclonus Syndrome" OR "Nocturnal Paroxysmal Dystonia" OR "REM Sleep Parasomnias +" OR "Restless Legs Syndrome" OR "Sleep Arousal Disorders +" OR "Sleep Bruxism" OR "Sleep-Wake Transition Disorders" OR "atypical sleep" OR "difficult sleeping" OR "difficulties in sleeping" OR "difficulty sleeping" OR "disorder during sleep" OR "disorders during sleep" OR "disturbance during sleep" OR "disturbance of sleep" OR "disturbances during sleep" OR "disturbances of sleep" OR "problem during sleep" OR "problem of sleep" OR "problems during sleep" OR "problems of sleep" OR "sleep disturbance" OR "sleep disturbances" OR "sleep interference" OR "sleep perturbation" OR "sleep perturbations" OR "sleep problem" OR "sleep problems" OR "sleep related disease" OR "sleep related disorder" OR "sleep related problem" OR "sleeping difficulties" OR "sleeping difficulty" OR "sleeping disorder" OR "sleeping disorders" OR "sleeping problem" OR "sleeping problems" OR "trouble sleeping" |
| #3 |  |
| Title/Abstract | "acupoint herbal patching" OR "acupoint application" OR "acupoint herbal plaster" OR "acupoint patch" OR "acupoint patching" OR "acupoint sticking" OR "point application" OR "point application therapy" OR "acupoint herbal patching" OR "AHP" OR "Chinese acupoint application" OR "herbal application of acupoint paste" OR "Xueweitiefu" OR "acupressure application" OR "Drug Acupoint Application" OR "Herbal Patch" OR "Acupoint Patch" OR "External Application Therapy" OR "Plaster Therapy" OR "acupoint plaster" OR "herbal patching" |
| #4 | #1 AND #2 AND #3 |

检索过程截图（Retrieval Process and Retrieval Query）

**#1**

**#2**

**#3**

**#4**

**2.2 Cochrane Library**

| 检索方式 | Advanced - Medical terms(MeSH)-Search manager |
| --- | --- |
| 检索字段 | MeSH、Title Abstract Keyword |
| 检索时段 | 建库-至今（2026-04-01） |
| #1 |  |
| Medical terms(MeSH) | Stroke |
| #2 |  |
| Title Abstract Keyword | "cerebrovascular accident" OR "Strokes" OR "Cerebrovascular Accidents" OR "Cerebral Stroke" OR "Cerebral Strokes" OR "Stroke, Cerebral" OR "Strokes, Cerebral" OR "Cerebrovascular Apoplexy" OR "Apoplexy, Cerebrovascular" OR "Vascular Accident, Brain" OR "Brain Vascular Accident" OR "Brain Vascular Accidents" OR "Vascular Accidents, Brain" OR "Cerebrovascular Stroke" OR "Cerebrovascular Strokes" OR "Stroke, Cerebrovascular" OR "Strokes, Cerebrovascular" OR "Apoplexy" OR "CVA" OR "CVAs" OR "Stroke, Acute" OR "Acute Stroke" OR "Acute Strokes" OR "Strokes, Acute" OR "Cerebrovascular Accident, Acute" OR "Acute Cerebrovascular Accident" OR "Acute Cerebrovascular Accidents" OR "Cerebrovascular Accidents, Acute" OR "Brain Infarction" OR "Brain Stem Infarctions +" OR "Cerebral Infarction +" OR "Hemorrhagic Stroke" OR "Ischemic Stroke" OR "Embolic Stroke" OR "Thrombotic Stroke +" OR "accident, cerebrovascular" OR "acute cerebrovascular lesion" OR "acute focal cerebral vasculopathy" OR "apoplectic stroke" OR "apoplexia" OR "blood flow disturbance, brain" OR "brain accident" OR "brain attack" OR "brain blood flow disturbance" OR "brain insult" OR "brain insultus" OR "cerebral apoplexia" OR "cerebral insult" OR "cerebral vascular accident" OR "cerebral vascular insufficiency" OR "cerebro vascular accident" OR "cerebrovascular arrest" OR "cerebrovascular failure" OR "cerebrovascular injury" OR "cerebrovascular insufficiency" OR "cerebrovascular insult" OR "cerebrum vascular accident" OR "cryptogenic stroke" OR "insultus cerebralis" OR "ischaemic seizure" OR "ischemic seizure" OR "cerebrovascular accident" |
| #3 | #1 OR #2 |
| #4 |  |
| Medical terms(MeSH) | Sleep Wake Disorders |
| #5 |  |
| Title Abstract Keyword | "sleep disorder" OR "Disorder, Sleep Wake" OR "Disorders, Sleep Wake" OR "Sleep Wake Disorder" OR "Wake Disorder, Sleep" OR "Wake Disorders, Sleep" OR "Sleep Disorders" OR "Disorder, Sleep" OR "Disorders, Sleep" OR "Long Sleeper Syndrome" OR "Long Sleeper Syndromes" OR "Sleeper Syndrome, Long" OR "Sleeper Syndromes, Long" OR "Syndrome, Long Sleeper" OR "Syndromes, Long Sleeper" OR "Short Sleeper Syndrome" OR "Short Sleeper Syndromes" OR "Sleeper Syndrome, Short" OR "Sleeper Syndromes, Short" OR "Syndrome, Short Sleeper" OR "Syndromes, Short Sleeper" OR "Short Sleep Phenotype" OR "Phenotype, Short Sleep" OR "Phenotypes, Short Sleep" OR "Short Sleep Phenotypes" OR "Sleep Phenotypes, Short" OR "Sleep-Related Neurogenic Tachypnea" OR "Neurogenic Tachypnea, Sleep-Related" OR "Neurogenic Tachypneas, Sleep-Related" OR "Sleep Related Neurogenic Tachypnea" OR "Sleep-Related Neurogenic Tachypneas" OR "Tachypnea, Sleep-Related Neurogenic" OR "Tachypneas, Sleep-Related Neurogenic" OR "Subwakefullness Syndrome" OR "Subwakefullness Syndromes" OR "Syndromes, Subwakefullness" OR "Syndrome, Subwakefullness" OR "Dyssomnias" OR "Sleep Deprivation" OR "Sleep Disorders, Circadian Rhythm +" OR "Sleep Disorders, Intrinsic +" OR "Parasomnias" OR "Nocturnal Myoclonus Syndrome" OR "Nocturnal Paroxysmal Dystonia" OR "REM Sleep Parasomnias +" OR "Restless Legs Syndrome" OR "Sleep Arousal Disorders +" OR "Sleep Bruxism" OR "Sleep-Wake Transition Disorders" OR "atypical sleep" OR "difficult sleeping" OR "difficulties in sleeping" OR "difficulty sleeping" OR "disorder during sleep" OR "disorders during sleep" OR "disturbance during sleep" OR "disturbance of sleep" OR "disturbances during sleep" OR "disturbances of sleep" OR "problem during sleep" OR "problem of sleep" OR "problems during sleep" OR "problems of sleep" OR "sleep disturbance" OR "sleep disturbances" OR "sleep interference" OR "sleep perturbation" OR "sleep perturbations" OR "sleep problem" OR "sleep problems" OR "sleep related disease" OR "sleep related disorder" OR "sleep related problem" OR "sleeping difficulties" OR "sleeping difficulty" OR "sleeping disorder" OR "sleeping disorders" OR "sleeping problem" OR "sleeping problems" OR "trouble sleeping" |
| #6 | #4 OR #5 |
| #7 |  |
| Title Abstract Keyword | "acupoint herbal patching" OR "acupoint application" OR "acupoint herbal plaster" OR "acupoint patch" OR "acupoint patching" OR "acupoint sticking" OR "point application" OR "point application therapy" OR "acupoint herbal patching" OR "AHP" OR "Chinese acupoint application" OR "herbal application of acupoint paste" OR "Xueweitiefu" OR "acupressure application" OR "Drug Acupoint Application" OR "Herbal Patch" OR "Acupoint Patch" OR "External Application Therapy" OR "Plaster Therapy" OR "acupoint plaster" OR "herbal patching" |
| #8 | #3 AND #6 AND #7 |

检索过程截图（Search process screenshot）

#1

#2

#3

#4

#5

#6

#7

#8

**2.3 Embase**

| 检索方式 | Search |
| --- | --- |
| 检索字段 | Emtree term - exploded、Title or Abstract |
| 检索时段 | 建库-至今（2026-04-01） |
| #1 |  |
| Emtree term - exploded | cerebrovascular accident |
| Title or Abstract | 'Stroke' OR 'Strokes' OR 'Cerebrovascular Accidents' OR 'Cerebral Stroke' OR 'Cerebral Strokes' OR 'Stroke, Cerebral' OR 'Strokes, Cerebral' OR 'Cerebrovascular Apoplexy' OR 'Apoplexy, Cerebrovascular' OR 'Vascular Accident, Brain' OR 'Brain Vascular Accident' OR 'Brain Vascular Accidents' OR 'Vascular Accidents, Brain' OR 'Cerebrovascular Stroke' OR 'Cerebrovascular Strokes' OR 'Stroke, Cerebrovascular' OR 'Strokes, Cerebrovascular' OR 'Apoplexy' OR 'CVA' OR 'CVAs' OR 'Stroke, Acute' OR 'Acute Stroke' OR 'Acute Strokes' OR 'Strokes, Acute' OR 'Cerebrovascular Accident, Acute' OR 'Acute Cerebrovascular Accident' OR 'Acute Cerebrovascular Accidents' OR 'Cerebrovascular Accidents, Acute' OR 'Brain Infarction' OR 'Brain Stem Infarctions +' OR 'Cerebral Infarction +' OR 'Hemorrhagic Stroke' OR 'Ischemic Stroke' OR 'Embolic Stroke' OR 'Thrombotic Stroke +' OR 'accident, cerebrovascular' OR 'acute cerebrovascular lesion' OR 'acute focal cerebral vasculopathy' OR 'apoplectic stroke' OR 'apoplexia' OR 'blood flow disturbance, brain' OR 'brain accident' OR 'brain attack' OR 'brain blood flow disturbance' OR 'brain insult' OR 'brain insultus' OR 'cerebral apoplexia' OR 'cerebral insult' OR 'cerebral vascular accident' OR 'cerebral vascular insufficiency' OR 'cerebro vascular accident' OR 'cerebrovascular arrest' OR 'cerebrovascular failure' OR 'cerebrovascular injury' OR 'cerebrovascular insufficiency' OR 'cerebrovascular insult' OR 'cerebrum vascular accident' OR 'cryptogenic stroke' OR 'insultus cerebralis' OR 'ischaemic seizure' OR 'ischemic seizure' OR 'cerebrovascular accident' |
| #2 |  |
| Emtree term - exploded | sleep disorder |
| Title or Abstract | 'Sleep Wake Disorders' OR 'Disorder, Sleep Wake' OR 'Disorders, Sleep Wake' OR 'Sleep Wake Disorder' OR 'Wake Disorder, Sleep' OR 'Wake Disorders, Sleep' OR 'Sleep Disorders' OR 'Disorder, Sleep' OR 'Disorders, Sleep' OR 'Long Sleeper Syndrome' OR 'Long Sleeper Syndromes' OR 'Sleeper Syndrome, Long' OR 'Sleeper Syndromes, Long' OR 'Syndrome, Long Sleeper' OR 'Syndromes, Long Sleeper' OR 'Short Sleeper Syndrome' OR 'Short Sleeper Syndromes' OR 'Sleeper Syndrome, Short' OR 'Sleeper Syndromes, Short' OR 'Syndrome, Short Sleeper' OR 'Syndromes, Short Sleeper' OR 'Short Sleep Phenotype' OR 'Phenotype, Short Sleep' OR 'Phenotypes, Short Sleep' OR 'Short Sleep Phenotypes' OR 'Sleep Phenotypes, Short' OR 'Sleep-Related Neurogenic Tachypnea' OR 'Neurogenic Tachypnea, Sleep-Related' OR 'Neurogenic Tachypneas, Sleep-Related' OR 'Sleep Related Neurogenic Tachypnea' OR 'Sleep-Related Neurogenic Tachypneas' OR 'Tachypnea, Sleep-Related Neurogenic' OR 'Tachypneas, Sleep-Related Neurogenic' OR 'Subwakefullness Syndrome' OR 'Subwakefullness Syndromes' OR 'Syndromes, Subwakefullness' OR 'Syndrome, Subwakefullness' OR 'Dyssomnias' OR 'Sleep Deprivation' OR 'Sleep Disorders, Circadian Rhythm +' OR 'Sleep Disorders, Intrinsic +' OR 'Parasomnias' OR 'Nocturnal Myoclonus Syndrome' OR 'Nocturnal Paroxysmal Dystonia' OR 'REM Sleep Parasomnias +' OR 'Restless Legs Syndrome' OR 'Sleep Arousal Disorders +' OR 'Sleep Bruxism' OR 'Sleep-Wake Transition Disorders' OR 'atypical sleep' OR 'difficult sleeping' OR 'difficulties in sleeping' OR 'difficulty sleeping' OR 'disorder during sleep' OR 'disorders during sleep' OR 'disturbance during sleep' OR 'disturbance of sleep' OR 'disturbances during sleep' OR 'disturbances of sleep' OR 'problem during sleep' OR 'problem of sleep' OR 'problems during sleep' OR 'problems of sleep' OR 'sleep disturbance' OR 'sleep disturbances' OR 'sleep interference' OR 'sleep perturbation' OR 'sleep perturbations' OR 'sleep problem' OR 'sleep problems' OR 'sleep related disease' OR 'sleep related disorder' OR 'sleep related problem' OR 'sleeping difficulties' OR 'sleeping difficulty' OR 'sleeping disorder' OR 'sleeping disorders' OR 'sleeping problem' OR 'sleeping problems' OR 'trouble sleeping' |
| #3 |  |
| Title or Abstract | 'acupoint herbal patching' OR 'acupoint application' OR 'acupoint herbal plaster' OR 'acupoint patch' OR 'acupoint patching' OR 'acupoint sticking' OR 'point application' OR 'point application therapy' OR 'acupoint herbal patching' OR 'AHP' OR 'Chinese acupoint application' OR 'herbal application of acupoint paste' OR 'Xueweitiefu' OR 'acupressure application' OR 'Drug Acupoint Application' OR 'Herbal Patch' OR 'Acupoint Patch' OR 'External Application Therapy' OR 'Plaster Therapy' OR 'acupoint plaster' OR 'herbal patching' |
| #4 | #1 AND #2 AND #3 |

检索过程截图（Search process screenshot）

#1

#2

#3

#4

**2.4 Web of Science**

| 检索方式 | Advanced - FIELDED SEARCH |
| --- | --- |
| 检索字段 | Topic |
| 检索时段 | 建库-至今（2026-04-01） |
| Topic | "cerebrovascular accident" OR "Stroke" OR "Strokes" OR "Cerebrovascular Accidents" OR "Cerebral Stroke" OR "Cerebral Strokes" OR "Stroke, Cerebral" OR "Strokes, Cerebral" OR "Cerebrovascular Apoplexy" OR "Apoplexy, Cerebrovascular" OR "Vascular Accident, Brain" OR "Brain Vascular Accident" OR "Brain Vascular Accidents" OR "Vascular Accidents, Brain" OR "Cerebrovascular Stroke" OR "Cerebrovascular Strokes" OR "Stroke, Cerebrovascular" OR "Strokes, Cerebrovascular" OR "Apoplexy" OR "CVA" OR "CVAs" OR "Stroke, Acute" OR "Acute Stroke" OR "Acute Strokes" OR "Strokes, Acute" OR "Cerebrovascular Accident, Acute" OR "Acute Cerebrovascular Accident" OR "Acute Cerebrovascular Accidents" OR "Cerebrovascular Accidents, Acute" OR "Brain Infarction" OR "Brain Stem Infarctions +" OR "Cerebral Infarction +" OR "Hemorrhagic Stroke" OR "Ischemic Stroke" OR "Embolic Stroke" OR "Thrombotic Stroke +" OR "accident, cerebrovascular" OR "acute cerebrovascular lesion" OR "acute focal cerebral vasculopathy" OR "apoplectic stroke" OR "apoplexia" OR "blood flow disturbance, brain" OR "brain accident" OR "brain attack" OR "brain blood flow disturbance" OR "brain insult" OR "brain insultus" OR "cerebral apoplexia" OR "cerebral insult" OR "cerebral vascular accident" OR "cerebral vascular insufficiency" OR "cerebro vascular accident" OR "cerebrovascular arrest" OR "cerebrovascular failure" OR "cerebrovascular injury" OR "cerebrovascular insufficiency" OR "cerebrovascular insult" OR "cerebrum vascular accident" OR "cryptogenic stroke" OR "insultus cerebralis" OR "ischaemic seizure" OR "ischemic seizure" OR "cerebrovascular accident" |
| AND Topic | "sleep disorder" OR "Sleep Wake Disorders" OR "Disorder, Sleep Wake" OR "Disorders, Sleep Wake" OR "Sleep Wake Disorder" OR "Wake Disorder, Sleep" OR "Wake Disorders, Sleep" OR "Sleep Disorders" OR "Disorder, Sleep" OR "Disorders, Sleep" OR "Long Sleeper Syndrome" OR "Long Sleeper Syndromes" OR "Sleeper Syndrome, Long" OR "Sleeper Syndromes, Long" OR "Syndrome, Long Sleeper" OR "Syndromes, Long Sleeper" OR "Short Sleeper Syndrome" OR "Short Sleeper Syndromes" OR "Sleeper Syndrome, Short" OR "Sleeper Syndromes, Short" OR "Syndrome, Short Sleeper" OR "Syndromes, Short Sleeper" OR "Short Sleep Phenotype" OR "Phenotype, Short Sleep" OR "Phenotypes, Short Sleep" OR "Short Sleep Phenotypes" OR "Sleep Phenotypes, Short" OR "Sleep-Related Neurogenic Tachypnea" OR "Neurogenic Tachypnea, Sleep-Related" OR "Neurogenic Tachypneas, Sleep-Related" OR "Sleep Related Neurogenic Tachypnea" OR "Sleep-Related Neurogenic Tachypneas" OR "Tachypnea, Sleep-Related Neurogenic" OR "Tachypneas, Sleep-Related Neurogenic" OR "Subwakefullness Syndrome" OR "Subwakefullness Syndromes" OR "Syndromes, Subwakefullness" OR "Syndrome, Subwakefullness" OR "Dyssomnias" OR "Sleep Deprivation" OR "Sleep Disorders, Circadian Rhythm +" OR "Sleep Disorders, Intrinsic +" OR "Parasomnias" OR "Nocturnal Myoclonus Syndrome" OR "Nocturnal Paroxysmal Dystonia" OR "REM Sleep Parasomnias +" OR "Restless Legs Syndrome" OR "Sleep Arousal Disorders +" OR "Sleep Bruxism" OR "Sleep-Wake Transition Disorders" OR "atypical sleep" OR "difficult sleeping" OR "difficulties in sleeping" OR "difficulty sleeping" OR "disorder during sleep" OR "disorders during sleep" OR "disturbance during sleep" OR "disturbance of sleep" OR "disturbances during sleep" OR "disturbances of sleep" OR "problem during sleep" OR "problem of sleep" OR "problems during sleep" OR "problems of sleep" OR "sleep disturbance" OR "sleep disturbances" OR "sleep interference" OR "sleep perturbation" OR "sleep perturbations" OR "sleep problem" OR "sleep problems" OR "sleep related disease" OR "sleep related disorder" OR "sleep related problem" OR "sleeping difficulties" OR "sleeping difficulty" OR "sleeping disorder" OR "sleeping disorders" OR "sleeping problem" OR "sleeping problems" OR "trouble sleeping" |
| AND Topic | "acupoint herbal patching" OR "acupoint application" OR "acupoint herbal plaster" OR "acupoint patch" OR "acupoint patching" OR "acupoint sticking" OR "point application" OR "point application therapy" OR "acupoint herbal patching" OR "AHP" OR "Chinese acupoint application" OR "herbal application of acupoint paste" OR "Xueweitiefu" OR "acupressure application" OR "Drug Acupoint Application" OR "Herbal Patch" OR "Acupoint Patch" OR "External Application Therapy" OR "Plaster Therapy" OR "acupoint plaster" OR "herbal patching" |

检索过程截图（Search process screenshot）
